# Recommendations from the Galician Oncological Society and the Galician Society of Nuclear Medicine for the use of ^177^Lu-PSMA-617 radioligand-therapy in prostate cancer

**DOI:** 10.1007/s12094-024-03662-7

**Published:** 2024-09-12

**Authors:** Ovidio Fernández Calvo, José Muñoz Iglesias, Estephany Abou Jokh Casas, Aura Molina-Díaz, Urbano Anido Herranz, Javier Casas Nebra, Lucía García-Bernardo, Sara Martínez-Breijo, Martín Lázaro-Quintela, Gloria Muñiz-García, Sergio Vázquez-Estevez

**Affiliations:** 1 Department of Medical Oncology, Complexo Hospitalario Universitario de Ourense, Ourense, Spain; 2 Department of Nuclear Medicine (SERGAS), University Hospital of Vigo, Meixoeiro Hospital, Vigo, Spain; 3 Department of Nuclear Medicine, Hospital Universitario Lucus Augusti, Lugo, Spain; 4 Department of Medical Oncology, Complexo Hospitalario Universitario de A Coruña, A Coruña, Spain; 5 Department of Medical Oncology, Complexo Hospitalario Universitario de Santiago de Compostela, Santiago de Compostela, Spain; 6 Uro-Oncology Unit, Complexo Hospitalario Universitario de A Coruña, A Coruña, Spain; 7 Department of Nuclear Medicine, Complexo Hospitalario Universitario de Santiago de Compostela, Santiago de Compostela, Spain; 8 Department of Urology, Complexo Hospitalario Universitario de A Coruña, A Coruña, Spain; 9 Department of Medical Oncology, University Hospital of Vigo, Meixoeiro Hospital, Vigo, Spain; 10 Department of Nuclear Medicine, Complexo Hospitalario Universitario de Ourense, Ourense, Spain; 11 Department of Medical Oncology, Hospital Universitario Lucus Augusti de Lugo, Lugo, Spain

**Keywords:** Theragnostic, Castration-resistant prostate cancer, Radioligands, PSMA, Recommendations

## Abstract

Theragnostic is a type of precision medicine that uses molecules linked to radioactive isotopes for the diagnosis and treatment of diseases. In recent years, it has gained significant importance to treat neuroendocrine tumors and is currently being used in prostate cancer. Various radiopharmaceuticals have emerged for diagnosing and detecting lesions showing prostate-specific membrane antigen (PSMA) positivity on the Positron emission tomography/computed tomography scan, being the most widely used labeled with [^68^Ga] and [^18^F]. Its use as therapy in prostate cancer (PC) has been assessed in the VISION, TheraP, and PSMAfore clinical trials conducted with the radioligand [^177^Lu]Lu-PSMA-617, demonstrating significant antitumor activity. The aim of this article is to present practical recommendations, based on current available scientific evidence and on a multidisciplinary consensus, for the diagnosis and treatment with [^177^Lu]Lu-PSMA-617 in patients with PC.

## Introduction

Theragnostic has been used in nuclear medicine since 1946 when Saul Hertz and Arthur Roberts first treated hyperthyroidism with [^131^I] at the Massachusetts General Hospital (Boston, MA, US) [1]. Several decades after, in 1998, John Funkhouser coined the term “theranostics” when he developed a test to monitor the effectiveness of a new anticoagulant drug [2].

The term “theragnostic” was coined to explain scientific advances aimed at establishing more specific and individualized therapies, uniting diagnostic and therapeutic applications with the same agent, resulting in a therapeutic approach that involves diagnosis and radioisotope therapy, and monitoring of treatment response [3].

For diagnostic purposes, radioisotopes emitting gamma radiation ([^99^^m^Tc], [^131^I], [^111^In]) or positrons ([^18^F], [^68^Ga]) are used, while radioactive isotopes ([^131^I], [^223^Ra], [^177^Lu], [^125^Ac]) emitting beta or alpha radiation are used for therapeutic reasons. These radioisotopes are attached to molecules or ligands, which can be peptides, proteins, antibodies, etc. The target cells to which these radioisotopes will bind can be membrane enzymes, antigens, hormone receptors, etc. The labeled compounds will follow different metabolic pathways, reflecting their biodistribution, affinity, and ligand concentration in the target tissue. If a high concentration is found after diagnosis, it can be used to treat that target tissue. Therefore, it is essential to confirm biodistribution for diagnosis and treatment to be similar. At the same time, they can be concentrated in other non-tumor tissues, such as salivary glands, intestinal mucosa, and proximal renal tubular cells, but always in lower concentration than in the target cells. There is a positive correlation between the level of ligand concentration and the severity of the target lesion [4, 5].

In recent decades, theragnostic has experienced significant growth, with its use in the management of neuroendocrine tumors, also currently being used in prostate cancer (PC). This is reflected in the publication of a set of guidelines on the needs and requirements of Theragnostic Units, involving the European Association of Nuclear Medicine (EANM), Society of Nuclear Medicine and Molecular Imaging (SNMMI), and International Atomic Energy Agency (IAEA) [6].

## Castration-resistant PC: current situation

Despite receiving curative intent treatment, the recurrence rate of PC is estimated at around 27–53% [7]. Once local therapies with radical intent have failed, many PC patients will stop responding to hormonal therapy, thus becoming patients with castration-resistant prostate cancer (CRPC).

It is a heterogeneous disease with multiple molecular changes, some of them due to the development of treatment resistance, mainly affecting the AR pathway (about 85%), the PI3K-AKT-mTOR pathway (about 40%), and others such as neuroendocrine differentiation. [8–10].

Determining factors for choosing CRPC treatment include previous treatments, response, and response time in the hormone-sensitive stage, identified genetic and molecular changes, histologic subtypes, and clinical parameters.

Since the publication of the TAX 327 trial in 2004, which demonstrated the efficacy profile of docetaxel as the first-line therapy of metastatic CRPC (mCRPC) [11], other therapeutic alternatives have been developed. These include hormonal agents targeting the AR, such as abiraterone, which demonstrated its efficacy profile in the pivotal COU-AA-301 and 302 studies [12, 13], and Enzalutamide in the PREVAIL and AFFIRM trials [14, 15]. Olaparib, in the PROFOUND trial [16], and rucaparib, in the TRITON 2 trial [17], demonstrated their efficacy profiles in patients with mCRPC and mutations in BRCA1, BRCA2, or ATM, as well as the combination of niraparib + abiraterone + prednisone in patients with homologous recombination repair gene changes in the MAGNITUDE trial [18]. The use of talazoparib as the first-line therapy of asymptomatic or mildly symptomatic patients with mCRPC on androgen deprivation therapy (TALAPRO-2 trial) [19] and abiraterone as the first-line therapy of patients with mCRPC (PROPEL trial) [20] have also been studied. A different therapeutic alternative is beta emitters such as [^177^Lu]Lu-PSMA-617, which demonstrated its efficacy profile in the VISION trial conducted with patients with a positive PSMA positron emission tomography (PET)/computed tomography (CT) scan [21]. In addition, the TROPIC trial demonstrated the efficacy and safety profile of the combination of cabazitaxel and prednisone in patients with mCRPC with disease progression after docetaxel [22]. Furthermore, the ALSYMPCA trial proved the efficacy profile of [^223^Ra] in patients with CRPC with bone metastases [23].

## Basics on theragnostic in PC

The Prostate-Specific Membrane Antigene (PSMA) is a type II cellular transmembrane glycoprotein with a dominant extracellular portion. Its expression is increased in PC cells compared to other cells, and its expression level has prognostic value regarding disease progression [24]. In addition, the uptake of PSMA-targeted radiopharmaceuticals in PET/CT scan correlates with degree tumor expression [24], making it an excellent target for theragnostic.

Regarding diagnosis, searching for lesions that show PSMA positivity through PET/CT scan, several radioligands are available, being [^68^Ga] Ga-PSMA-11 and [^18^F]F-DCFPyL the most common of all. These radioligands have demonstrated a similar efficacy profile [25, 26] and have been approved by the Food and Drug Administration (FDA) and European Medicines Agency (EMA) [27, 28].

The results of the VISION trial [21] conducted with [^177^Lu]Lu-PSMA-617 led to its approval by the FDA in March 2022 and the EMA in December 2022. Therefore, [^177^Lu]Lu-PSMA-617, along with androgen deprivation therapy (ADT) with or without AR pathway inhibitors, is indicated to treat adult patients with progressive PSMA-positive mCRPC treated with AR pathway inhibitors and taxane chemotherapy [29].

[^177^Lu] decays by emitting beta-negative radiation (78%) and gamma radiation (11%). Its physical half-life is 6.65 days. Once the radioligand binds to PSMA-expressing tumor cells, the beta-negative emission of [^177^Lu] irradiates the target cell and its surroundings, causing DNA damage and cell death. The mean penetration of [^177^Lu] into tissue is 0.67–2 mm, resulting in minimal damage to surrounding tissue [29].

The biodistribution of [^177^Lu]Lu-PSMA-617 shows uptake in the lacrimal glands, salivary glands, kidneys, bladder, liver, and small intestine (left and right colon). It does not undergo hepatic. It is eliminated through renal excretion with an elimination half-life of 41.6 h. The most common side effect is xerostomia, usually occurring in a mild degree [29].

The highest absorbed doses are detected in the lacrimal glands and salivary glands, but they remain below accepted levels, considering the criteria of ICRP publication 103 [30] even in repeated treatment cycles [31]. Various dosimetric trials with both [^177^Lu]-DOTA-TATE and [^177^Lu]Lu-PSMA-617 have demonstrated the feasibility of outpatient treatment [32]. Both therapies show a high excretion rate in the 1st h after administration. Approximately 50% of the activity administered to the patient is excreted through the kidneys after 4 h. At 4–6 h, patients present a dose rate lower or equal to 30 µSv/h at 1 m, being within the limits accepted by the regulations of many countries as discharge criteria [6].

Given the expected high demand for these treatments, their administration on an outpatient basis in the Theragnosis Units could facilitate the increase of their performance and at the same time reduce the discomfort of patients by avoiding their hospitalization in isolation in the Theragnosis Units.

## PSMA-base imaging theragnostics evidence in PC

The diagnostic role of PSMA PET/CT scan has been extensively studied in various scenarios of PC as described in the jointly published guidelines by the EANM and SNMMI [33], as well as in the consensus document on appropriate use criteria for PSMA PET scan [25].

According to the evidence currently available, its use is recommended in the initial staging of unfavorable intermediate and high-risk PC, in recurrence and biochemical persistence, in nmCRPC based on conventional imaging modalities, and in patient selection for potential PSMA-targeted therapy [33].

While there may be small differences between each PSMA radioligand ([^68^Ga]Ga-PSMA-11 and [18F]F-DCFPyl), there is currently no evidence of better diagnostic precision between them. A meta-analysis [34] revealed that the rate of recurrence detection is quite similar among the most widely used PSMA radiopharmaceuticals, and it is also much higher than conventional imaging modalities and other PET/CT scan radiopharmaceuticals such as choline or fluciclovine, which are commonly used.

In the initial staging, an adequate assessment of the extent of the disease is fundamental for the choice of the most appropriate therapeutic plan for each patient. Although imaging studies are not indicated in low and very low-risk patients due to the low probability of extraprostatic extension. Based on the evidence, PSMA PET/CT does seem to be appropriate for the initial staging of unfavorable intermediate, high, or very high-risk PC, being superior to conventional imaging.

In most studies, high specificity and predictive values stand out. Thus, in the prospective multicenter Phase-3 trial that led to the approval of the radiopharmaceutical [^68^Ga]Ga-PSMA-11, Hope et al. [35] found that in 277 patients with high-risk PC with PET/CT with [^68^Ga]Ga-PSMA-11 before prostatectomy, 27% of patients had a positive result for pelvic lymph node metastases with a sensitivity of 40%, a specificity of 95%, a positive predictive value of 75% and a negative predictive value of 81%. Similarly, in the OSPREY [36] trial, a prospective multicenter trial that led to FDA approval of the fluorinated radiopharmaceutical ([^18^F]F-DCFPyl), 252 patients undergoing radical prostatectomy were analyzed, and a specificity of 97.9% and a low sensitivity of 40.3% were found for the detection of pelvic lymph nodes. The positive predictive value was 86.7% and the negative predictive value 83.2%. However, in a post hoc analysis, which took into account lymph nodes > 5 mm, it was seen that the specificity remained at 98%, but the sensitivity rose from 40 to 60%, and the NPV also increased to 93%.

Some studies have compared the performance of PSMA PET/CT versus conventional imaging. The proPSMA trial stands out, in which 302 patients with high-risk PCa were analyzed and in which they wanted to compare conventional imaging techniques versus [^68^Ga]Ga-PSMA-11 PET/CT, demonstrating that PSMA PET had a higher efficacy in diagnosis (92% versus 65% for TIC), higher sensitivity (85% versus. 38%), as well as a greater impact on treatment, fewer equivocal results (7% versus. 23%), lower radiation exposure, greater agreement in reports, and no adverse events secondary to PSMA were seen [37].

In another study of 160 high-risk PC patients evaluated with [^18^F]F-DCFPyl PET/CT for initial staging, 90% of patients with distant metastases were correctly identified, with 48% of these patients having no enlarged lymph nodes on CT. Additional metastatic lymph nodes were detected in almost all patients (41/42), and [^18^F]F-DCFPyl PET altered patient management in 17% of patients [38].

Early detection of biochemical recurrence (BCR) after radical treatment (radical prostatectomy or radiotherapy) allows effective treatment to be offered before the appearance of clinical recurrence. Conventional imaging techniques (bone scintigraphy, CT and MRI) have a low diagnostic yield in asymptomatic patients with low PSA levels [39], and the sensitivity of PET/CT with choline derivatives falls to suboptimal values in patients with a low PSA level (< 1 ng/mL) [40].

There is enough evidence of the usefulness of PSMA PET in the context of BCR, including prospective studies with [^68^Ga]Ga-PSMA-11 and [^18^F]F-DCFPyl, which have demonstrated high detection rates with a high positive predictive value, being superior to TIC, and impacting therapeutic planning and patient prognosis.

Perera et al. [41] found in a meta-analysis that included 4790 patients that PET with [^68^Ga]Ga-PSMA-11 had high detection rates, being higher with increasing PSA levels, even 33% for PSA levels below 0.19 ng/ml and 95% for PSA levels > 2.0 ng/mL. This study found a high sensitivity and specificity in the detection of lymph node metastases in patients with BCR (75% and 99%, respectively), being much higher than those of conventional imaging techniques [39].

Similarly, in another meta-analysis by Hope et al., they found a sensitivity of 99% and a specificity of 76% in the detection of pelvic lymph nodes in patients with BCR using histopathological correlation as the gold standard [42].

PSMA PET/CT appears substantially more sensitive than choline PET/CT at any PSA value, but especially for PSA levels ≤ 1, where the PSMA detection rate is doubled [40].

Furthermore, in a recent systematic review and meta-analysis, Pozdnyakov et al. [43] found that PSMA PET modified treatment plans in 56.4% and achieved a BCR-free survival of 60.2% at a median follow-up of 20 months, consistent with other previous trials [44, 45].

Another diagnostic use of PSMA PET is the identification of patients with mCRPC that is PSMA-positive, for whom PSMA-targeted therapy is indicated.

The FDA approval of [^177^Lu]Lu-PSMA-617 as radioligand therapy (RLT) for the management of progressive mCRPC [46] was simultaneously accompanied by the approval of the diagnostic radiopharmaceutical [^68^Ga]Ga-PSMA-11, to assess the eligibility of this treatment in these patients [27].

The efficacy of [^68^Ga]Ga-PSMA-11 for this indication is based on the VISION study [21], where PET/CT with Gallium (^68^Ga) gozetotide was used to identify patients with positive PSMA. It should be noted that although other diagnostic PSMA radiopharmaceuticals do not have approval in the technical data sheet for this indication, they can be used interchangeably given the similarity in diagnostic accuracy demonstrated between them [34].

## Current clinical evidence of theragnostics in the management of mCRPC

The VISION trial was the first multicenter, open-label, randomized phase-III trial that included patients with mCRPC progressing after, at least, one androgen receptor pathway inhibitor (ARPI) and one or two taxane regimens (Table 1), with high PSMA uptake [21]. The study primary endpoint was to determine the radiographic progression-free survival (rPFS) and overall survival (OS). Patients were randomized on a 2:1 ratio to receive [^177^Lu]Lu-PSMA-617 plus SOC (study group), or SOC alone (control group) [47, 48], which was determined by the investigator, excluding the use of chemotherapy, immunotherapy, [^223^Ra], and investigational drugs. A total of 831 patients were included in the study, 551 of whom were assigned to the study group and 280 to the control group.

**Table 1:** Phase-III clinical trials on [^177^Lu]Lu-PSMA-617 therapy

Study	VISION	TheraP	PSMAfore
Type of study	Phase 3 Randomized 2:1 Multicenter international Open-label	Phase 2 Randomized 1:1 Multicenter Open-label	Phase 3 Randomized 1:1 Multicenter international Open-label
No. of patients	Assessed: 1179 Randomized: 831 (82.9%) Study arm: 511 Control arm: 280	Assessed: 291 Randomized: 200 Study arm: 99 Control arm: 101	Assessed: 585 Randomized: 468 Study arm: 227 Control arm: 232
Population	mCRPC treated with ≥ 1 ARSI and 1 or 2 taxanes	mCRPC progressing to docetaxel and eligible for cabazitaxel	CPRCm progressing to ARSI, taxane-naive
Primary endpoints	OS rPFS	PSA50 response	rPFS
Secondary endpoints	ORR (RECIST 1.1) CDR Time to first SSE Safety and tolerability PSA Response HRQoL and pain	OS rPFS PFS PSA HRQoL	OS PSA50, SBE PFS- Soft tissue HRQoL, safety, tolerability ORR, DCR, DOR PFS PSA, PFS-pain Biomarkers
Study arm	^177^Lu-PSMA-617 7.4 GB/cycle up to six cycles + Protocol allowed by SOC	^177^Lu-PSMA-617 8.5 GBq IV every 6 weeks up to six cycles	^177^Lu-PSMA-617 7.4 GB/cycle c6sem up to six cycles
Control arm	Protocol allowed by SOC	Cabazitaxel (20 mg/m2 every 3 weeks up to ten cycles)	Hormone switch (abiraterone/enzalutamide/apalutamide)
Inclusion criteria	PSMA uptake > liver (SUVmax > 5–7) ECOG 0–2 Life expectancy > 6 months	Disease progression with PSA levels > 20. Proper renal, hematologic, and hepatic function. ECOG 0–2 PSMA lesions + with SUVmax > 20 and negative FDG	> 1 PSMA-positive metastatic lesion. Progression to a second-generation hormone. Taxane-naïve Ineligible for PARP inhibitors ECOG 0–1
Exclusion criteria	Patients with PSMA-negative lesions. Previous therapies with chemotherapy, immunotherapy, radium-223, investigational drugs	FDG-positive and PSMA-negative lesions (miss match)	Previous therapies with radiopharmaceuticals, chemotherapy, immunotherapy. Lesions in the central nervous system, spinal cord compression
Crossover	No	NA	Yes
PET scan/CT performed	^68^Ga-PSMA-11 PET scan/CT Centralized reporting	^68^ Ga-PSMA PET scan/CT ^18^F-FDG PET scan/CT	^68^ Ga-PSMA-11 PET scan/TC
Median of cycles of ^177^Lu-PSMA received	5	5	-
SPECT/CT after each ^177^Lu-PSMA cycle	No	Yes	Optional
Imaging screening failure	126 (12.6%)	91 (28%)	47 (7.7%), pending article publication
Median follow-up	20.9 months	18.4 months	15.9 months
mPFS	8.7 versus 3.4 months (*n* = 581) HR, 0.4 (99.2%CI 0.29–0.57) p < 0.001	At 12 months: 19% (95%CI 12–27) versus 3% (1–9)	12 months versus 5.59 months HR, 0.43; CI 95% 0.33–0.54 *p* > < 0.0001
mOS	15.3 versus 11.3 months (*n* = 831) HR, 0.62 (CI 95%, 0.52–0.74) *p* < 0.001	At 35.7 months: 19.1 m (16–9-21.4) versus 19.6 m (17.4–21.8)	Non-adjusted crossover (84.2%): 12.72 versus 13.08 months (HR, 1.16; CI95%) Adjusted crossover (84.2%): 12.72 versus 13.08 months (HR, 0.80; CI95%)
(RECIST 1.1)	ORR: 29.8% versus 1.7% DCR: 89% versus 66.7% CR: 9.2% versus 0% PR: 41.88% versus 3%	ORR: 49% versus 24% [95% CI 33–65 y 11–38]	ORR: 50.7% (38.6–62.8) versus 14.9% (7.7–25.0) DCR: 13.63 (11.56-NA) versus 10.05 (4.63-NA)
Rate of PSA response > 50%	46% versus 7.1%	66% versus 37%; *p* < 0.0001	57.6% versus 20%
Toxicity of the trial arm versus the control arm	Grade 3–4: 52.7% versus 38.0%	Grade 3–4: 33% versus 53%	Grade 3–4: 33.9 versus 43.1%

*DCR* Disease control rate, *mOS* median overall survival, *mPFS* median progression-free survival, *ORR* Objective response rate, *SBE* Symptomatic bone event

After a median follow-up of 20.9 months, it was demonstrated that [^177^Lu]Lu-PSMA-617 + SOC improved rPFS significantly with a median of 5.3 months (8.7 v. 3.4 months; HR, 0.40; *p* < 0.001) and OS by 4 months (15.3 versus 11.3 months; HR, 0.62; *p* < 0.001) compared to the control group [8]. There was also a 60% reduction in the risk of progression or death (HR, 0.40) [48]. At the same time, a favorable radiologic response was observed in the study group, with a CR in 9.2% versus 0%, and a PR in 41.8% versus 3% of the patients. Similarly, the time to first symptomatic bone event or death was longer in the study group, 11.5 months (95% CI 10.3–13.2) versus 6.8 months (95%CI 5.2–8.5) in the control group (HR, 0.50; *p* < 0.001) [24]. Grade 3 or 4 adverse events were more common in the study group, including anemia (15% versus 6%), lymphopenia (51% versus 19%), and thrombocytopenia (9% versus 2%) [49]. Despite the favorable results of the VISION trial, we should mention the high initial patient dropout rate (56%) of the control group, which was subsequently compensated, bringing it down to 16% [50].

The TheraP is a multicenter phase II trial that compared the efficacy and safety profile of [^177^Lu]Lu-PSMA-617 with cabazitaxel in patients with mCRPC progressing after docetaxel, being the primary endpoint the determination of the PSA50 response, defined as the number of patients with a ≥ 50% reduction in the PSA since treatment (50,53). A total of 98 patients received [^177^Lu]Lu-PSMA-617, and 85 were on cabazitaxel. The [^177^Lu]Lu-PSMA-617 treatment group had a higher PSA50 response rate compared to the control group (66% versus 37%, *p* < 0.0001), a longer rPFS rate (49% versus 24%; HR, 0.63; *p* = 0.0028) and fewer Grade 3 or 4 adverse events (33% versus 53%) [51]. Data from the secondary analysis of OS with a median follow-up of 35.7 months have recently been published. The OS was similar among those assigned to [177Lu]Lu-PSMA-617 versus those assigned to cabazitaxel (19.1 versus 19.6 months; *p* = 0.77) [52].

Recently, the results of the PSMAfore trial were released [53], a phase III, prospective, randomized, multicenter trial, with the primary endpoint of determining rPFS with [^177^Lu]Lu-PSMA-617 treatment versus a change in antiandrogenic treatment (abiraterone, enzalutamide) in taxane-naive mCRPC patients with PSMA-positive disease progressing on a second-generation hormonal treatment (ARPI). A total of 468 patients were randomized, 227 of whom received [^177^Lu]Lu-PSMA-617 treatment while 232 remained in the control arm. Secondary endpoints include OS, PSA50, symptom-free survival, objective response rate (ORR), and disease control rate (DCR), among others.

The study was positive the primary endpoint was achieved, with an rPFS of 12.02 months in the [^177^Lu]Lu-PSMA-617 arm versus 5.59 months in the ARPI change arm, after a median follow-up of 15.9 months (HR, 0.43; 95% CI 0.33–0.54; *p* < 0.0001). This was accompanied by results favorable to the [^177^Lu]Lu-PSMA-617 arm in terms of ORR, DCR, PSA50, and HRQoL. There were no statistically significant differences in the still immature OS analysis between the two study arms (HR, 1.16, 95% Confidence Intervals [CI] 0.83–1.64), being OS = 19.25 months (95%CI, 16.95-NE) in patients treated with [^177^Lu]Lu-PSMA-617 versus 19.71 months (95%CI, 17.81-NE) in the control arm, likely influenced by the fact that an 84.2% of patients who discontinued ARPI therapy due to radiographic progression crossed over to the ^177^Lu-PSMA-617 arm. These results slightly improve after statistical adjustment for the control arm crossover, but they remain immature, and final OS results are awaited.

The RALU trial evaluated the feasibility of the sequential use of alpha and beta emitter therapies in the context of bone-predominant mCRPC. Patients received a median of six cycles of [^223^Ra], and 59% of the patients a median of four cycles of [^177^Lu]Lu-PSMA. The median OS was 12.6 months (95%CI 8.8–16.1) and 31.4 months (95%CI 25.7–37.6) from the start of [^177^Lu]Lu-PSMA or [^223^Ra], respectively. After a 30-day follow-up, the most common grade 3–4 adverse events were anemia (18%) and thrombocytopenia (2%) [54].

The treatment with [^177^Lu]Lu-PSMA-617 was approved by the EMA on 09/12/2022 in combination with ADT with or without androgen receptor (AR) pathway inhibition is indicated for the treatment of adult patients with progressive PSMA-positive mCRPC who have been treated with AR pathway inhibition and taxane-based chemotherapy [29].

### Current therapeutic positioning

**Fig. 1:**
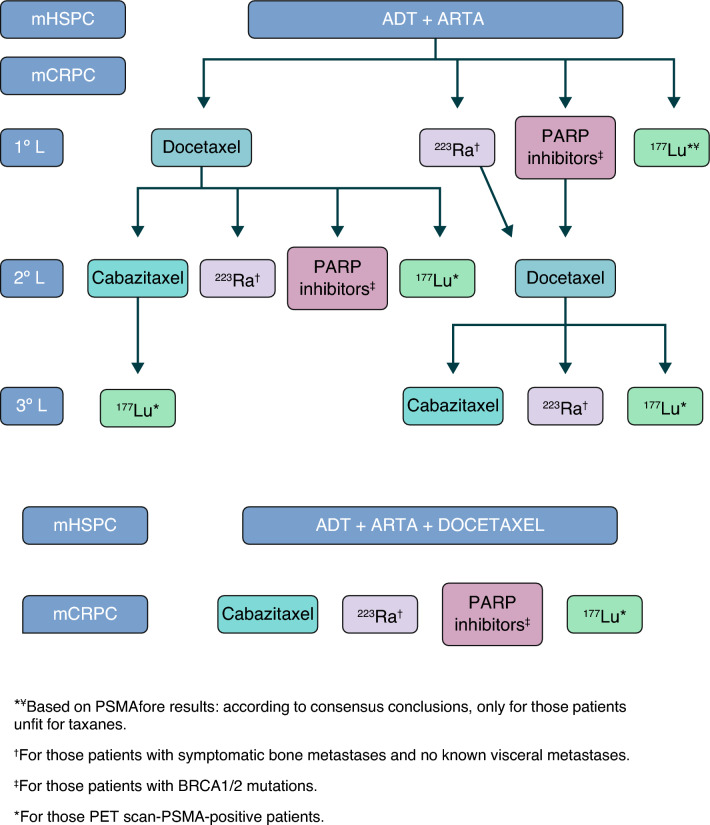
Current therapeutic positioning. *¥ Based on PSMAfore results: according to consensus, Only for those patients unfit for taxanes. †For those patients with symptomatic bone metastases and no known visceral metastases. ‡For those patients with BRCA1/2 mutations. *For those PET scan-PSMA-positive patients

Figure 1 illustrates the therapeutic algorithm proposed by the authors of this consensus.

## Patient profile

### Defining a positive PSMA PET/CT scan in the mCRPC setting

In the consensus published by the SNMMI [26], it is indicated that PSMA PET/CT scan should be performed to confirm the expression of the target receptor in tumor lesions since RLT will only be effective in “PSMA-positive” lesions and assess the likelihood of response to it. The two randomized trials that evaluated treatment selection patients based on PSMA expression through PET/CT scan but used different criteria to define PSMA PET/CT scan positivity.

The Phase-III VISION trial [21] required higher uptake than in the hepatic parenchyma in all measurable lesions based on visual assessment. A measurable lesion was defined as the detection of lymph nodes ≥ 2.5 cm of short-axis diameter, solid organ metastases ≥ 1 cm in short-axis diameter, and bone metastases with a soft-tissue component ≥ 1 cm in short-axis diameter. Patients with PSMA-negative disease (measurable lesions with uptake equal to or less compared to the liver) were also excluded from the study. Although there is limited evidence of clinical benefit in patients who do not meet the VISION trial criteria, in a series of patients who did not meet imaging criteria, the median OS was 9.6 months, and PSA50 response was 21%, lower than the 15 months and 46%, respectively, in the [^177^Lu]Lu-PSMA-617 treated cohort of the VISION trial [55].

The Phase II TheraP trial [56] required that, at least, one lesion should have a SUVmax > 20, and all measurable lesions a SUVmax > 10. Furthermore, it excluded patients with FDG PET scan positive/PSMA-negative disease (PSMA-negativity defined as SUVmax < 10).

The TheraP trial criteria resulted in a higher failure rate in the imaging screening compared to that reported in the VISION trial (28% versus 13%, respectively). A secondary analysis of the study showed that patients with higher PET/CT scan uptake also had a higher PSA response rate to [^177^Lu]Lu-PSMA-617 treatment, although patients with low PSMA uptake had higher PSA response rates with [^177^Lu]Lu-PSMA-617 than with cabazitaxel.

This working group considers the following recommendations for patient selection:

- It is recommended to implement the criteria used in the VISION trial (higher uptake compared to the liver) for patient selection purposes, as these criteria produced an OS benefit in the largest cohort of patients. Preferably, the baseline PSMA PET/CT scan should be performed within 3 months before treatment, as it is important for the baseline PSMA PET/CT scan to reflect the current status of the disease. If there is evidence of disease progression, it is recommended to repeat the test when possible.
- Both radiotherapeutics labeled with [^68^Ga] and [^18^F] can be used, although only [^68^Ga] PSMA-11 has an approved indication in its label for identifying patients with progressive PSMA-positive mCRPC for whom PSMA-targeted therapy is indicated. Although [^68^ Ga]Ga-PSMA-11 was used in the VISION and TheraP trials, the PSMA radiotherapeutics available have similar performances in prospective clinical studies, and in practice, they are considered equivalent tracers [34, 57].
- In addition to PSMA PET/CT scan, both the EANM and SNMMI [6, 24] recommend performing conventional imaging modalities (contrast-enhanced CT, MRI, or bone scintigraphy) to identify possible PSMA-negative disease, which is especially important in patients with known liver disease (strong recommendation).
- [^18^F]F-FDG PET/CT scan is not necessary as a standard tool for patient selection, although its use may be considered for further disease characterization if a patient exhibits signs of disease aggressiveness or there is suspicion of PSMA-negative disease. A conversion from PSMA-positive to negative phenotype has been described in liver metastases, and especially in this case, an additional [^18^F]F-FDG PET scan could be beneficial (weak recommendation) [26, 58].
- In addition, in certain cases, patients showing heterogeneous disease on the PSMA PET/CT scan could be treated. In cases with few PSMA-negative lesions, it might be appropriate to treat the dominant PSMA-positive disease using PSMA RLT. Since the criteria of the VISION trial define PSMA-negative disease in solid organs as ≥ 1 cm, the smaller volume PSMA-negative disease can be considered for treatment, especially if the disease is predominantly PSMA-positive. This could fundamentally be applied to patients who have exhausted all therapeutic options available to them.

The most widely accepted primary criterion for considering a PSMA-positive PET/CT scan result is the standardized uptake value in the tumor, which should be higher than that in the liver, as defined in the VISION trial [21]. In a post hoc analysis of this trial, higher PSMA PET scan uptake was associated with better outcomes; however, patients in the lowest quartile of expression also appeared to benefit compared to the control group [44]. In both the TheraP trial [51] and the VISION trial, higher uptake was associated with a better response [59].

Therefore, patients who may be eligible for treatment with ^177^Lu-PSMA-617 could include the following:

1. Patients with mCRPC with a positive PSMA PET/CT scan and who have progressed to, at least, one new hormonal treatment, and one or two taxane regimens.
2. Patients ineligible for chemotherapy after moving on to new hormonal agents, based on a Phase 2 randomized trial with 40 patients in which it appears to be non-inferior to docetaxel but still better tolerated [60]. It may also be considered if significant toxicity has developed with chemotherapy.

These types of drugs should be avoided (contraindicated) in the presence of myelosuppression and when creatinine clearance is < 30 mL/min. In patients with chronic kidney disease and no alternative therapeutic options, the risks of [^177^Lu]Lu-PSMA-617 toxicity and tumor progression should be assessed.

According to the EANM/SNMMI guidelines on [^177^Lu]Lu-PSMA-RLT therapy, the following conditions could represent relative contraindications: a life expectancy < 3 months, ECOG scores ≥ 3, poorly controlled urinary incontinence, acute obstruction of the urinary tract and/or unresolved obstructive uropathy, severe cardiac comorbidities, multiple organ failure or risk of multiple organ failure, acute infections, myelosuppression (WBC < 2.5/nL, ANC < 1.5/nL, Platelets < 75/nL).

## Imaging follow-up of PSMA-targeted therapy

There is currently no consensus on the use of PSMA PET/CT scan to assess response during treatment. Although it may be more accurate than gamma images performed after cycles for the visualization of PSMA-positive disease, there is no evidence that its use improves the management of the patient.

The physical characteristics of [^177^Lu] (beta and gamma emitter) allow visualization of the administered therapy, acquiring gamma images between 1 and 2 days after treatment (planar and/or SPECT). It also allows the visualization of changes in the extent of PSMA-positive disease after each cycle, making it a method for disease monitoring [58]. It has been demonstrated that changes in post-treatment gamma imaging, between the 1st and 2nd cycles, correlate with patient outcomes. A recent study [61] has shown that an increase in the calculated total tumor volume on the post-treatment SPECT/CT (1st and 2nd cycles) predicts a short PFS and may play a future role as an imaging response biomarker, identifying when to discontinue or intensify [^177^Lu]Lu-PSMA-617 therapy. In addition, it might play a role in the detection of residual disease after the 6th cycle and determine the need for additional therapies.

Regarding conventional imaging, in the VISION trial, patients were monitored with bone scintigraphy and CT scans every 12 weeks according to the protocol. The use of bone scintigraphy is considered optional and is primarily used to establish a new reference value after a good response to therapy or to confirm progression or response if there are any doubts based on the clinical or biochemical findings. Contrast-enhanced CT should be used to monitor treatment response, especially in cases with visceral and soft-tissue disease.

## Future

Despite the available evidence on this new therapeutic option, the best scenario for its use, possible combinations with other treatments, sequencing, retreatment, etc., still need to be elucidated. In future, radioligand therapy should explore new radionuclides such as Bismuth-213 [213Bi], Actinium-225 [^225^Ac], and Lead-212 [^212^Pb], all of which are alpha-emitters that could be added to the therapeutic armamentarium against PC. Biologically, these elements increase the number of double-strand DNA breaks, leading to cell death. Their potential drawback could be the production of daughter radioisotopes that would increase the half-life and may increase toxicity. Table 2 shows the main trials that will help determine how and when these therapies should be used.

**Table 2:** Clinical trials in the pipeline regarding prostate cancer with RLT

Neoadjuvant treatment for localized disease	^177^Lu-PSMA-617 (LuTectomy)
Locally advanced disease	^177^Lu-PSMA-617 + External beam radiation therapy (PROQURE-1)
First-line HSPC	^177^Lu-PSMA-617 (UpFrontPSMA - Docetaxel-, PSMAddition - ARPI-)
First-line mCRPC	^177^Lu-PSMA-I&T (BullsEye) ^177^Lu-PSMA-617 (PSMAfore) ^177^Lu-PSMA-617 + Enzalutamide (Enza-P)
Second-line mCRPC	^177^Lu-PSMA-617 (VISION, TheraP, Lu-PSMA) ^177^Lu-PSMA-617 + Cabazitaxel (LuCAB) ^177^Lu-PSMA-617 + Olaparib (LuPARP) ^177^Lu-PSMA-617 + Pembrolizumab (PRINCE) ^177^Lu-PSMA-617 + Ipilimumab-Nivolumab (EVOLUTION) ^177^Lu-PSMA-617 + Radium (ALPHABET) ^177^Lu-PSMA-617 + Abemaciclib (UPLIFT) ^177^Lu-PSMA-I&T (SPLASH, ECLIPSE) ^225^Ac-PSMA-617 (AcTION) ^177^Lu-PSMA-R2 (NCT03490838) ^227^Th-PSMA-TTC (NCT03724747) ^131^I-MIP-1095 + Enzalutamide (ARROW) 177Lu-J591 (NCT005338668) ^225^Ac-J591 (NCT045065567)

Two other relevant trials, even in the near future, are the PSMAddition (NCT04720157) [62] and ECLIPSE (NCT05204927). The PSMAddition is a Phase-III trial that will study the combination of [^177^Lu]-PSMA-617 and NHT (apalutamide, enzalutamide, or abiraterone) and ADT versus NHT-TDA as first-line therapy in mHSPC, being rPFS the primary endpoint. The ECLIPSE, which is similar to the previously mentioned PSMAfore, is another Phase-III trial that will compare [^177^Lu]-PSMA-I&T versus NHT (abiraterone or enzalutamide) to treat mCRPC that has progressed into a single line of NHT. Patients who have received chemotherapy before or who may have mutations in the DNA repair pathway and have not received treatment with olaparib or rucaparib will be excluded from the study. The study primary endpoint is also rPFS.

On the other hand, treatment individualization strategies based on dosimetry could improve the results of these treatments, considering the possibility of retreatment with alpha-emitters after progression to [^177^Lu]Lu-PSMA-617. In addition, the combination of radionuclide therapy bound to peptide receptors with PARP inhibitors could increase DNA double-strand breakage, while also preventing the repair of these breaks, theoretically improving the efficacy of both [63]. The combination with anti-androgens or androgen synthesis inhibitors could increase PSMA expression [64]. At present, the trials currently being conducted with olaparib (LuPARP), enzalutamide (Enza-P), docetaxel (UpFrontPSMA), pembrolizumab (PRINCE), or even radium-223 (ALPHABET), along with [^177^Lu]Lu-PSMA-617, and their results could improve our understanding of these combinations.

The LuTectomy is a prospective Phase I/II trial of dosimetry, safety, and potential benefit of treatment with [^177^Lu]Lu-PSMA-617 before surgery (PR and lymphadenectomy) in high-risk patients with localized de novo disease [65]. The results showed a partial PSMA response rate of 55%, stable disease in 40% of the patients, and progression in 5%. The median PSA reduction was 49%, and the PSA50 response rate was 45%. After a median follow-up of 13.8 months, the rate of biochemical PFS was 80%.

## Care continuum

**Fig. 2:**
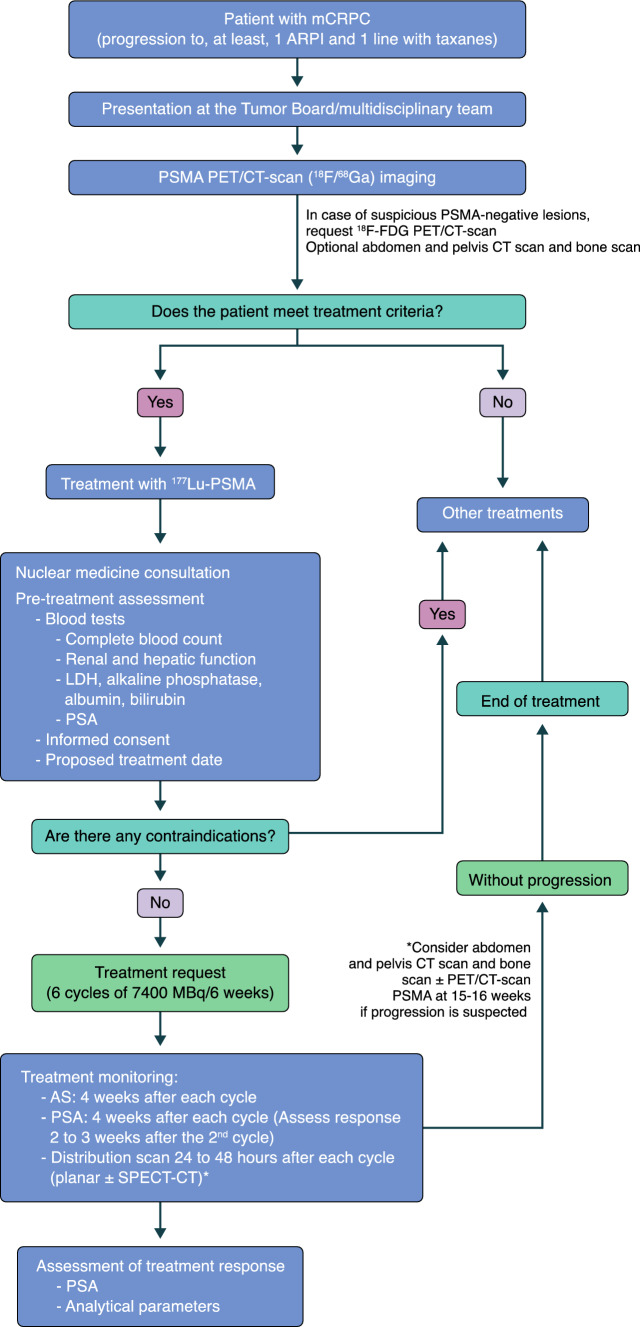
Proposal of care for patients with mCRPC

Based on current-existing recommendations, the multidisciplinary team proposes this care continuum (Fig. 2).

Morphologic response criteria in solid tumors have been the basis for assessing treatment response in clinical trials. Nearly two decades ago, the PCWG2 response criteria [66] were introduced, which, in addition to morphologic findings, included bone-scan findings, whose latest update (PCWG3) was published in 2016 [67].

The arrival of next-generation molecular imaging modalities, especially PSMA PET/CT scan, has revolutionized the management of patients with PC due to their impact on both staging and BCR [68]. Despite their proven superiority, their role in assessing treatment response still has not been elucidated [26, 58].

Several response criteria have recently been proposed that include PSMA PET scan-CT and are as follows:

- “Response evaluation criteria in PSMA PET/CT scan” (RECIP) to assess the efficacy profile of PSMA-ligand treatment in patients with mCRPC, developed in a multicenter retrospective trial at three academic medical centers, considering not only the number of new lesions but also changes in tumor volume [69]. This criterion has demonstrated higher accuracy compared with Response Evaluation Criteria in Solid Tumors 1.1 (RECIT 1.1), PCWG3 criteria; PET Response Criteria in Solid Tumors (PERCIST); and PSMA PET Progression (PPP), criteria for response evaluation using PSMA PET/CT. Nevertheless, they require validation in a prospective cohort.
- “EANM and EAU consensus document,” based on international experts in PC within the fields of nuclear medicine, radiology, and urology. Recommending the use of PSMA PET/CT scan to assess therapy response only when a change in clinical management is expected, categorizing patients as responders (CR, PR, and SD) and non-responders (DP) based on the appearance of new lesions and/or tumor volume [70].
- “PSMA PET/CT scan progression criteria” (PPP), based on expert recommendations and on the same principles as were applied for the PCGW criteria (including the assessment of biochemical or clinical progression), but adds value by including PSMA PET-CT [71].

## Discussion

Theragnostic is a specific and personalized therapy that involves the use of molecules attached to radioactive isotopes for the diagnosis and treatment of various diseases, using alpha or beta radiation from different radioisotopes. The cellular targets can be hormone receptors, enzymes, or membrane proteins, such as PSMA, where high expression and biodistribution need to be confirmed before their use.

Regarding the diagnosis and detection of PSMA-positive lesions using PET/CT scan, several radioligands remain available, being the most common ones labeled with [^68^Ga] and [^18^F], showing no significant differences between them. PSMA PET/CT scan has changed the current landscape of patients with PC, not only due to its impact on staging and localizing recurrences but also because it has been suggested as an essential tool for selecting patients with mCRPC who are eligible for radioligand therapy. However, although the PSMA PET/CT scan superiority over conventional imaging modality has been demonstrated, its role in assessing treatment response is still to be elucidated.

From a therapeutic perspective, the efficacy and safety profile of [^177^Lu]Lu-PSMA has been studied in the mCRPC setting in various prospective studies, at different points in the therapeutic sequence, primarily in two areas: patients who have received prior taxane therapy and those who have not. The results from the VISION trial conducted with [^177^Lu]Lu-PSMA-617 in which included patients with mCRPC progressing to, at least, 1 ARPI and 1 or 2 taxane regimens with high-PSMA uptake, demonstrate a higher PFS and OS compared to the SOC allowed. In the TheraP trial, [^177^Lu]Lu-PSMA-617 showed a higher response rate than cabazitaxel with a lower rate of side effects and similar OS data.

In the case of taxane-naive patients with mCRPC, the PSMAfore trial [38] included patients who had progressed to a prior ARPI and who were randomized to receive ^177^Lu-PSMA-617 or a hormonal therapy sequence with a change in ARPI. The results show a benefit in the primary endpoint of PFS, as well as in other secondary endpoints such as ORR and PSA50 reduction. We should mention the significant crossover rate (84.2%) of patients included in the sequential ARPI-to-^177^Lu-PSMA-617 arm after radiologic progression, conditioning the fact that favorable OS results have not yet been achieved, although these are still immature. We should mention that the chosen comparator arm is not a standard practice in Spain, and patients could have been better selected through stricter imaging criteria based on the SUVmean (SUVmean ≥ 10, for example). Therefore, we do not consider these results representative of a change in the SOC in this scenario, which still is docetaxel, or [^223^Ra], except for patients ineligible to receive taxanes.

## Conclusions

[^177^Lu]Lu-PSMA therapy has demonstrated efficacy in PFS and OS in patients with mCRPC refractory to docetaxel and/or cabazitaxel and better PFS in patient’s naïve to docetaxel. Furthermore, due to the improvement in quality of life, it should be considered as part of the usual therapeutic arsenal for these patients. Management of patients with this type of therapy must be multidisciplinary.

## Data Availability

No applicable.
